# Supplementary material for: CurveCurator: a recalibrated F-statistic to assess, classify, and explore significance of dose–response curves
Source: Nat Commun. 2023 Nov 30;14:7902. doi: 10.1038/s41467-023-43696-z (PMC10689459; doi:10.1038/s41467-023-43696-z)
Supplement: Supplementary file 1 — Supplementary Information [file 41467_2023_43696_MOESM1_ESM.pdf]

Supplementary Information for

CurveCurator: A recalibrated F-statistic to assess, classify, and explore significance of dose-response curves

*Florian P. Bayer, Manuel Gander, Bernhard Kuster, Matthew The**

** Corresponding Author*

This PDF contains:

- Supplementary figures S1 to S6.

- Supplementary notes

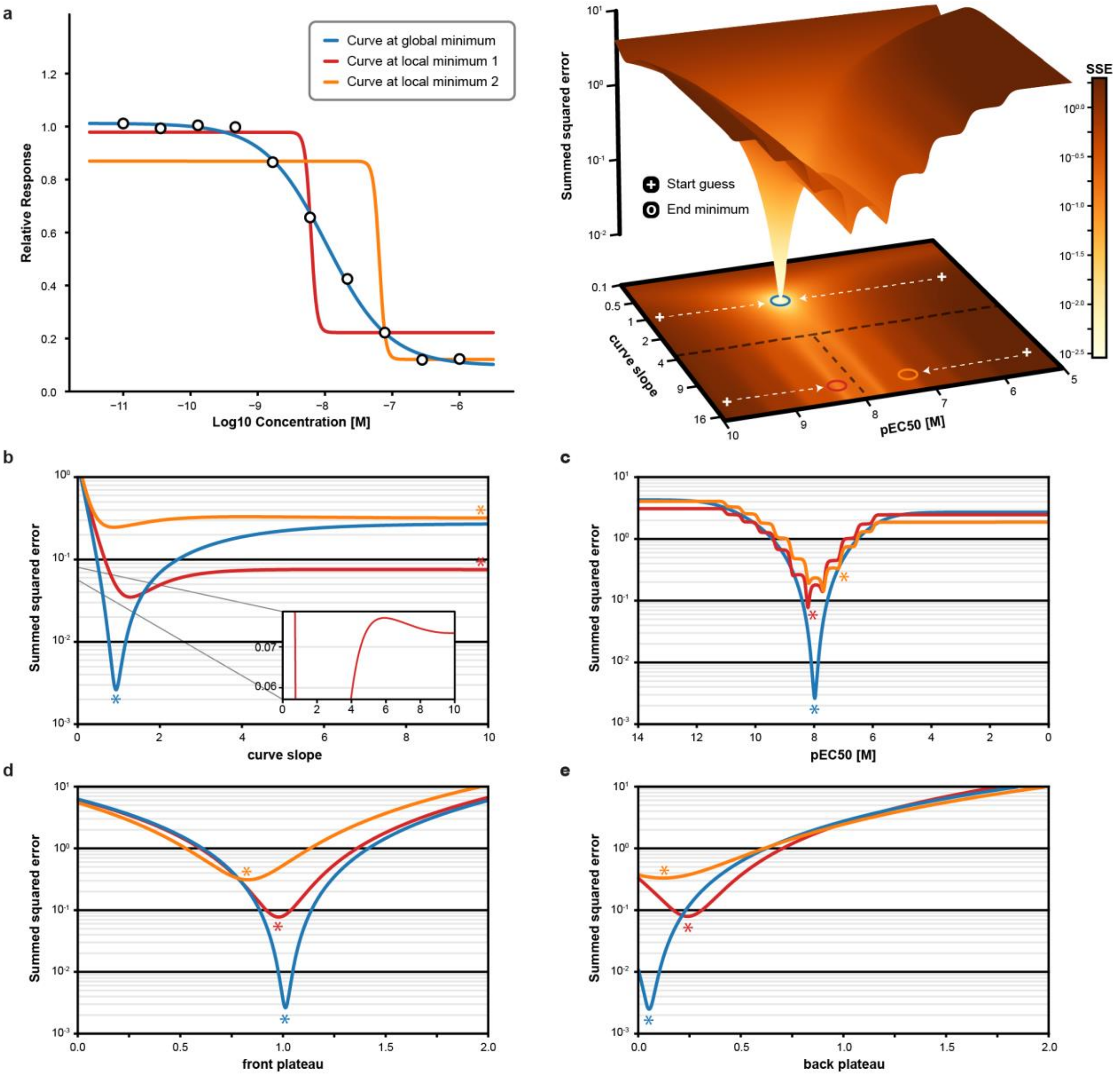
a
Curve at global minimum
Curve at local minimum 1
Curve at local minimum 2
Relative Response
Log10 Concentration [M]
Summed squared error
Start guess
End minimum
SSE
curve slope
pEC50 [M]
b
c
curve slope
pEC50 [M]
d
e
front plateau
back plateau

Fig S1. The minimization surface of the 4-parameter log-logistic function is not convex, leading to undesired convergence of curve fits to local minima.

a) *A dose-response curve was simulated (left panel; black data points, n=10) by adding random error to a down-going, ground-truth 4-parameter log-logistic model (pEC_{50}=8.0, slope=1.0, front plateau=1.0, back plateau=0.1). Three different initial guesses for the four parameters were tested, leading to three different curve fits (red, orange, and blue). Each initial guess converged to a different minimum, of which only one converged to the global minimum (blue) to achieve the best possible fit. The cost surface of slope vs. pEC_{50} is obtained from ordinary least squares for the same simulated dose-response curve (right panel). The color from white to brown indicates the summed squared error at one particular slope-pEC_{50} pair (white: good fit; brown: poor fit). Initial guesses are represented as white pluses on the 2D projection, and the arrows indicate the simplified trajectories from those initial guesses to the next minima (circles) during the minimization process. Two initial guesses converge to local minima (red and orange circle), whereas the other initial guess converges to the global minimum (blue circle).*

b-e) *One-dimensional cost plots based on the three minima (red, orange, and blue curves) from panel a when changing only one parameter at a time (slope, pEC_{50}, front plateau, back plateau) while fixing the other curve parameters with the values of the respective minimum (indicated by the asterisks). It shows that for the slope and pEC_{50} parameters, the cost functions are non-convex (multiple minima), and thus, the curve fit algorithm can get trapped in these local minima. The front and back plateau parameters have convex cost functions instead and thus will always converge to the locally optimal parameter value for any combination of the fixed parameters.*

Source data for panels a-e are provided as a Source Data file.

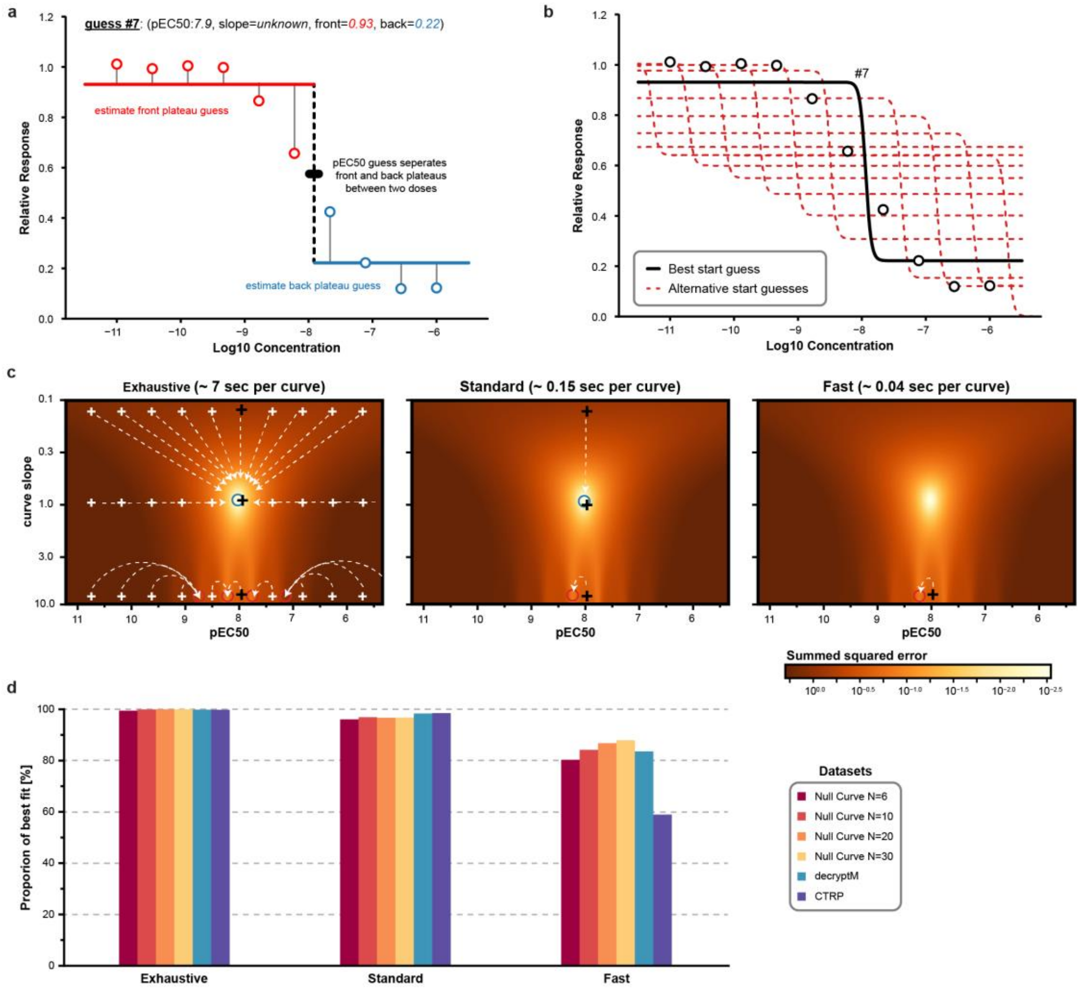
a
guess #7: (pEC50:7.9, slope=unknown, front=0.93, back=0.22)
estimate front plateau guess
pEC50 guess seperates front and back plateaus between two doses
estimate back plateau guess
Relative Response
Log10 Concentration
b
#7
Best start guess
Alternative start guesses
Relative Response
Log10 Concentration
c
Exhaustive (~ 7 sec per curve)
Standard (~ 0.15 sec per curve)
Fast (~ 0.04 sec per curve)
curve slope
pEC50
Summed squared error
d
Proporion of best fit [%]
Exhaustive
Standard
Fast
Datasets
Null Curve N=6
Null Curve N=10
Null Curve N=20
Null Curve N=30
decryptM
CTRP

Fig S2. Three strategies for setting initial parameter guesses lead to different success rates in reaching the global minimum.

a) *CurveCurator generates multiple initial guesses calculated from the measured response values (circles). The guessing procedure is exemplified for the start guess #7. Here, the pEC_{50} value is between data points 6 and 7 at approximately 12.5 nM. The data points are then divided into a front (<pEC_{50}, red) and back section (>pEC_{50}, blue), and the mean is used to calculate the plateau guess values, respectively. The slope is yet undetermined.*

b) *The cost of each possible start guess (1 - 11, red dashed lines) is evaluated by ordinary least squares to determine the best guess (black solid line), which happens to be guess #7 from panel a.*

c) *CurveCurator implemented three different minimization strategies (exhaustive, standard, fast) that use different numbers of start guesses to increase the odds of reaching the global minimum. The position of these start guesses on the cost surface is depicted with white crosses. The best start guesses are shown as black crosses. Note that the cost surface will look different for each dose-response curve, and this particular surface describes the curve from FigS1. "Exhaustive" uses all alternative start guesses with three different slopes and is, consequently, the slowest method and scales with N doses. Many start guesses converge to the same few minima (dotted white lines). "Standard" is more efficient and uses only the best guess with three different slopes. "Fast" only uses the best initial guess with a single arbitrary high slope value. The color from white to brown indicates the summed squared error at one particular slope-pEC_{50} pair (white: good fit; brown: poor fit).*

d) *We evaluated how often the global minimum was reached by the three strategies (exhaustive, standard, fast) for different numbers of doses (n = 6, 10, 20, 30) in simulated null curves as well as in real data sets, e.g., decryptM (Dasatinib in K562) and CTRP cell viability data. The global minimum was defined as the best F-statistic with an error tolerance of 0.1%. The standard strategy turns out to be the best compromise between time and fitting success.*

Source data for panels b and d are provided as a Source Data file.

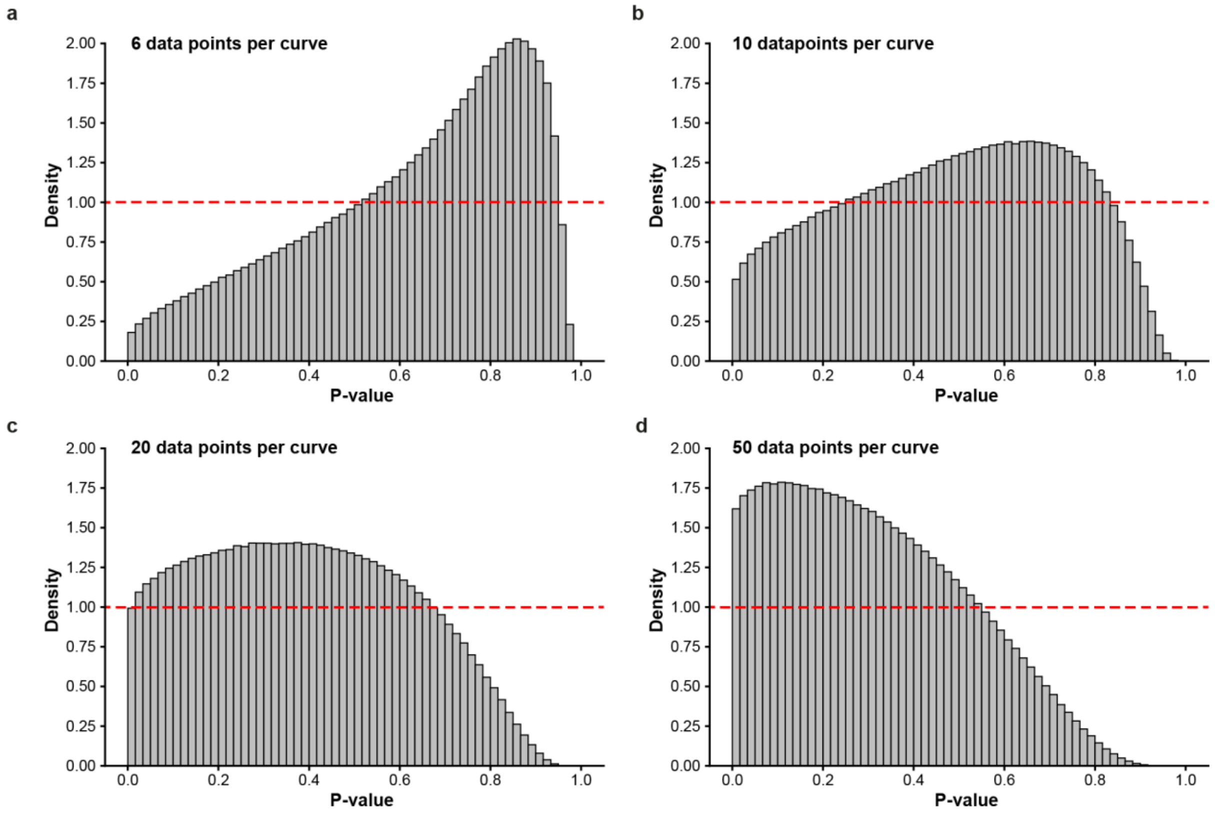

a
6 data points per curve
Density
P-value
b
10 datapoints per curve
c
20 data points per curve
d
50 data points per curve

Fig S3. P-value distributions for curves under the null hypothesis are not well calibrated using the standard F-statistic.

To analyze the statistical properties of dose-response curves, we simulated 5 million dose-response curves under the null hypothesis that the response is independent of the dose (no dose-dependent changes) and calculated p-values based on the classical F-statistic approach for linear models. We performed these simulations for ***a)*** *n = 6 doses,* ***b)*** *n = 10 doses,* ***c)*** *n = 20 doses, and* ***d)*** *n = 50 doses. Well-calibrated procedures should produce a uniform p-value distribution with a density of 1.0 (red dashed lines in all panels). For simulated data with few doses (n = 6 or n = 10), p-values are generally too high (conservative). For simulated data with many doses (n = 20 or n = 50), p-values are generally too low (anti-conservative).*

Source data for panels a-d are provided as a Source Data file.

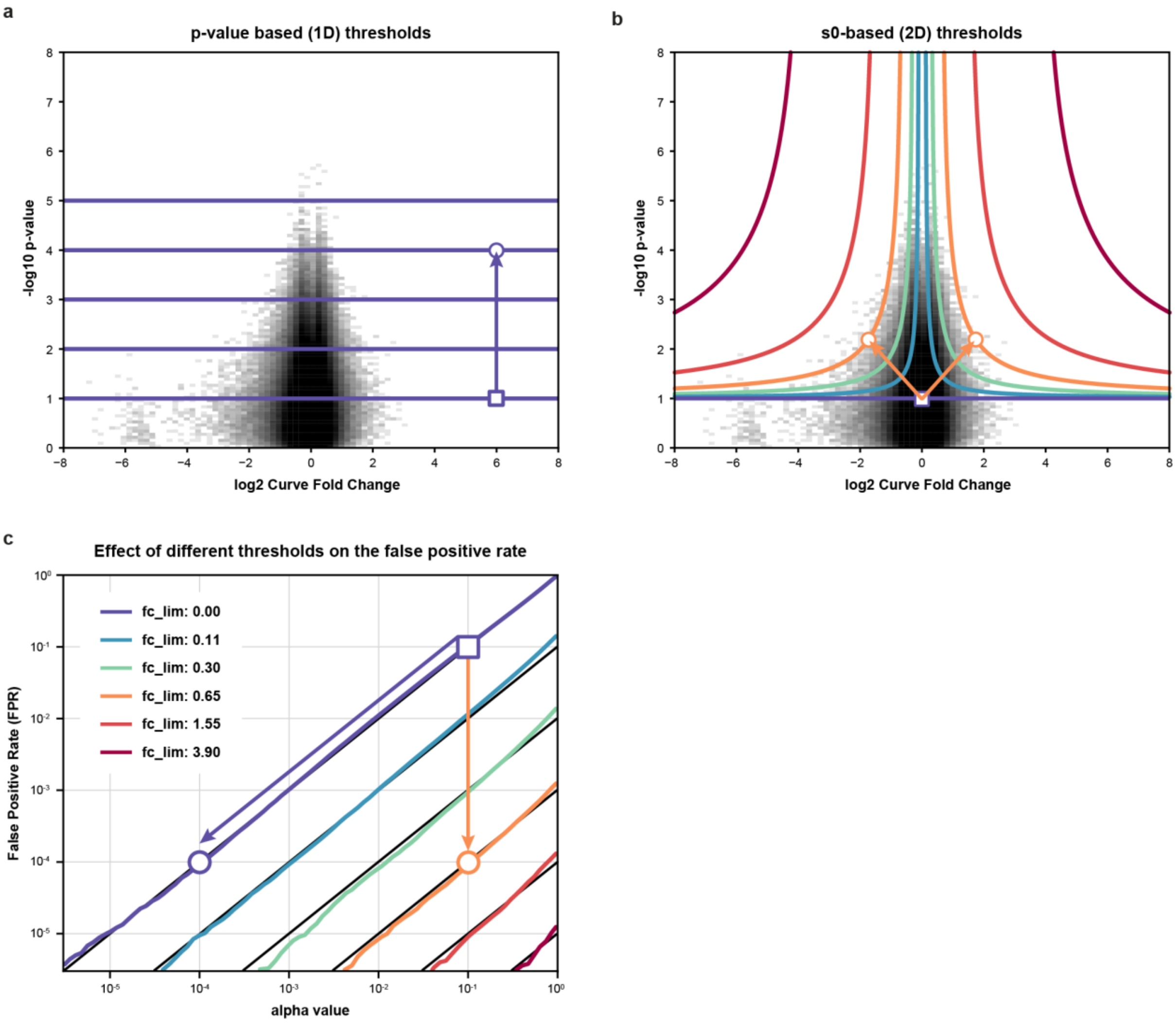
a
p-value based (1D) thresholds
-log10 p-value
log2 Curve Fold Change
b
s0-based (2D) thresholds
-log10 p-value
log2 Curve Fold Change
c
Effect of different thresholds on the false positive rate
False Positive Rate (FPR)
alpha value
fc_lim: 0.00
fc_lim: 0.11
fc_lim: 0.30
fc_lim: 0.65
fc_lim: 1.55
fc_lim: 3.90

Fig S4. Determining the significance of dose-response curves using the s_0 principle leads to an acceptance of a greater number of biologically relevant curves at the same FPR.

a) *Volcano plot (significance vs. effect size) of 5 million simulated null hypothesis dose-response curves (n=11 doses). Random curves with high significance tend to have small effect sizes. Traditional decision boundaries consider only the p-value (horizontal lines), where a user-defined alpha threshold (purple lines) establishes the false-positive rate (FPR). Lowering the FPR (box-to-circle arrow) can only be achieved by setting a more stringent alpha threshold, likely losing many true positives.*

b) *Same volcano plot as in panel a, but showing several hyperbolic decision boundaries using the SAM s_0 principle. These colored hyperbolic decision boundaries combine a particular fold change asymptote with a fixed significance asymptote of alpha = 0.1 and reject curves with small effect sizes but high significance. For example, the orange decision boundary has the same FPR as a traditional alpha threshold of 1e-4 but would accept biologically relevant dose-response curves with, e.g., a p-value of 1e-3 and a fold change of 2.0, which would have been rejected by the traditional alpha threshold.*

c) *For a fixed value of the fold-change asymptote, there is a linear relationship between the value of the alpha asymptote and the observed FPR. Consequently, the same FPR can be achieved by multiple combinations of fold change and alpha asymptotes. The purple arrow indicates a reduction of the FPR to 1e-4 by reducing the alpha threshold, as depicted in panel a. The orange arrow indicates the same reduction of the FPR by introducing a fold-change asymptote but keeping the alpha asymptote at 0.1, as depicted in panel b.*

Source data for panels a-c are provided as a Source Data file.

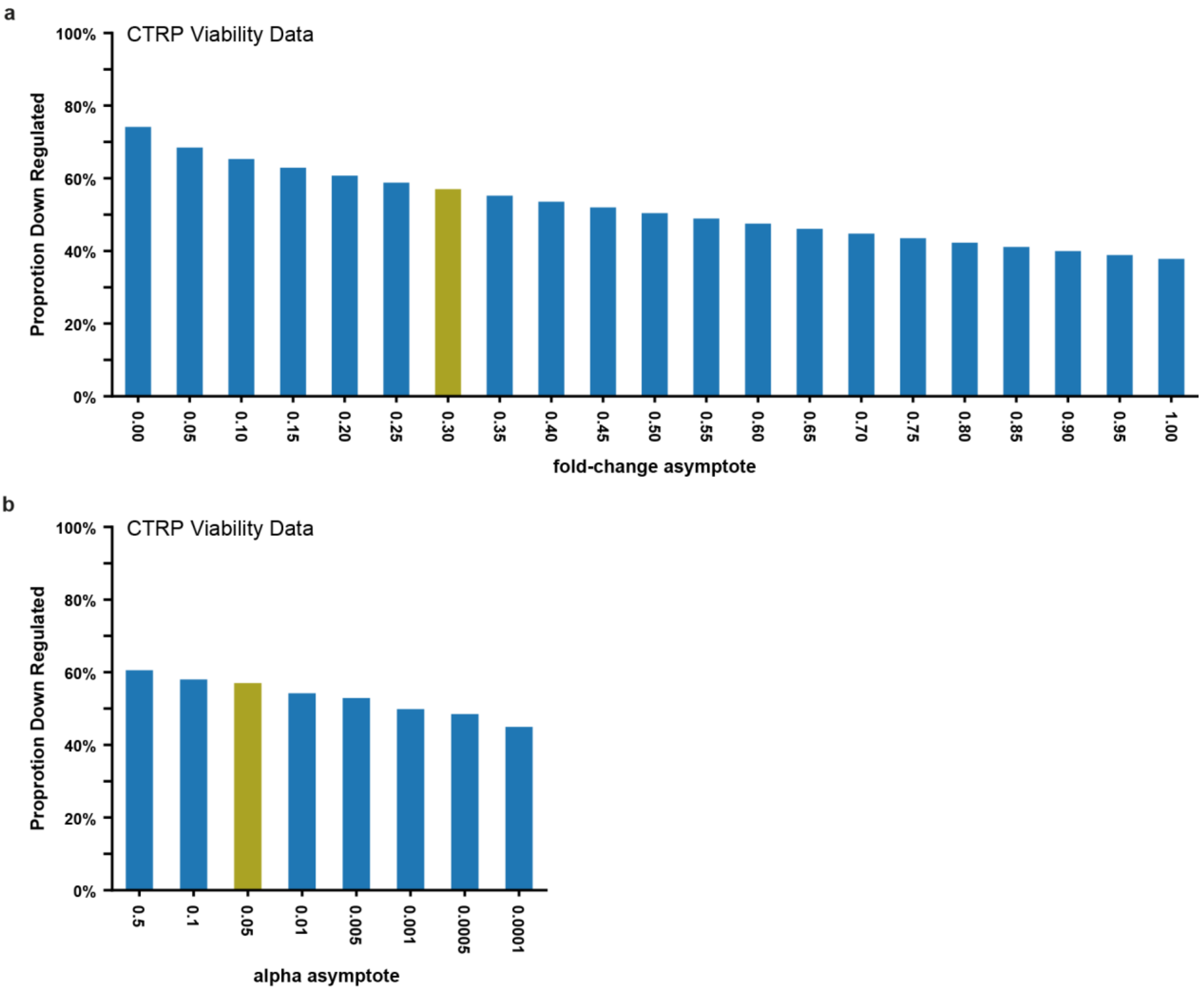
a
CTRP Viability Data
Proprotion Down Regulated
100%
80%
60%
40%
20%
0%
0.00 0.05 0.10 0.15 0.20 0.25 0.30 0.35 0.40 0.45 0.50 0.55 0.60 0.65 0.70 0.75 0.80 0.85 0.90 0.95 1.00
fold-change asymptote
b
CTRP Viability Data
Proprotion Down Regulated
100%
80%
60%
40%
20%
0%
0.5 0.1 0.05 0.01 0.005 0.001 0.0005 0.0001
alpha asymptote

Fig S5. Fold-change and alpha asymptotes define robust decision boundaries.

a) *CTRP viability data was reprocessed using different fold-change asymptotes and a constant alpha asymptote of 5%. In the bar plot, the proportion of down-regulated curves in the entire data set is plotted for each decision boundary. The yellow bar indicates the same decision boundary as in panel b.*

b) *Same plot as in panel a, but this time, the fold-change asymptote was fixed at 0.3, and different alpha asymptotes were used. The yellow bar indicates the same decision boundary as in panel a.*

Source data for panels a-b are provided as a Source Data file.

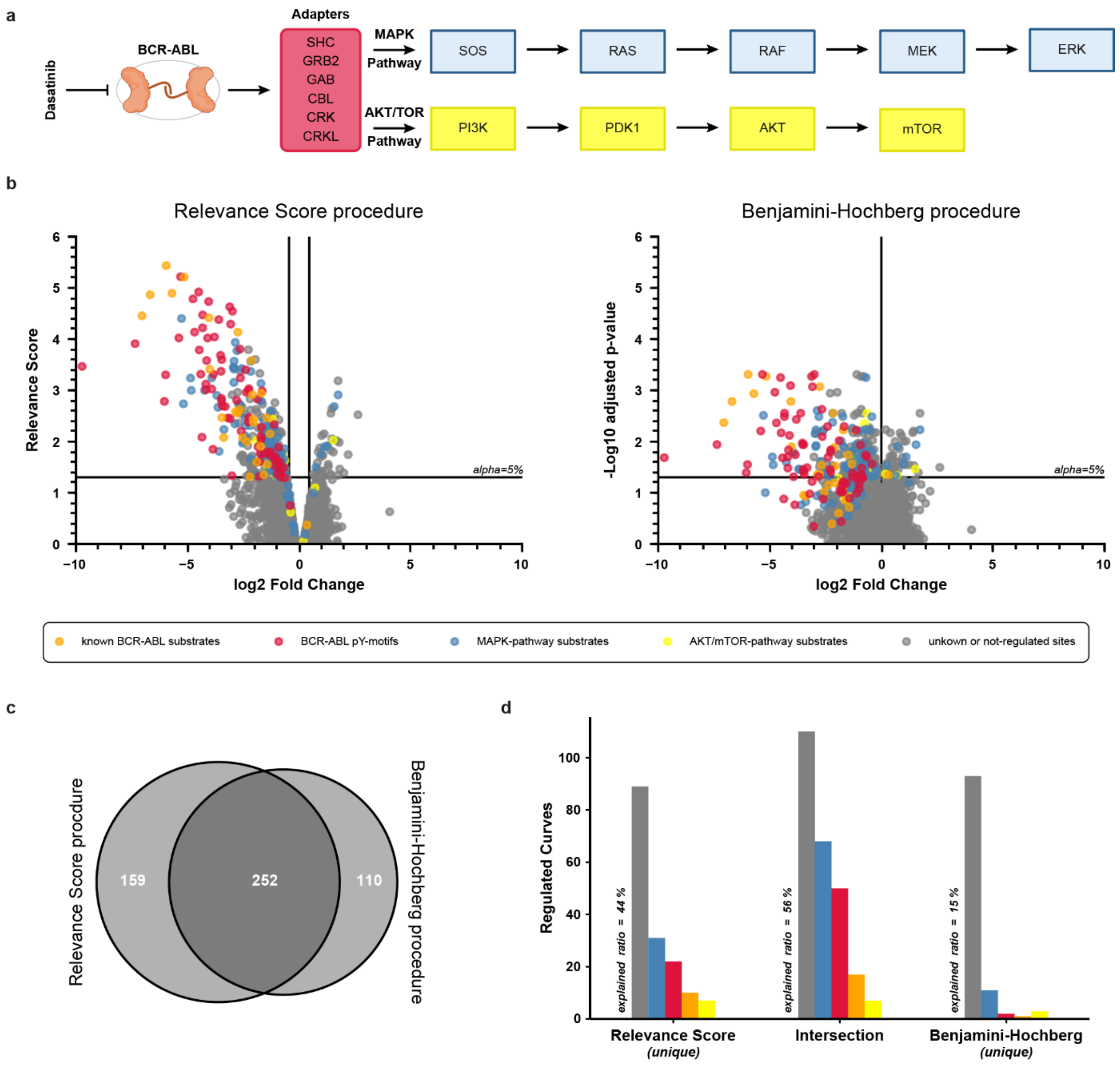
a
Dasatinib
BCR-ABL
Adapters
SHC
GRB2
GAB
CBL
CRK
CRKL
MAPK Pathway
AKT/TOR Pathway
SOS
RAS
RAF
MEK
ERK
PI3K
PDK1
AKT
mTOR
b
Relevance Score procedure
Benjamini-Hochberg procedure
Relevance Score
-Log10 adjusted p-value
log2 Fold Change
alpha=5%
known BCR-ABL substrates
BCR-ABL pY-motifs
MAPK-pathway substrates
AKT/mTOR-pathway substrates
unkown or not-regulated sites
c
Relevance Score procdure
Benjamini-Hochberg procedure
159
252
110
d
Regulated Curves
explained ratio = 44 %
explained ratio = 56 %
explained ratio = 15 %
Relevance Score (unique)
Intersection
Benjamini-Hochberg (unique)

Fig S6. The relevance score retains more biologically important phosphorylation sites

a) *Simplified signaling network of K562 upon Dasatinib perturbation. Due to the connectivity of kinases and the pivotal role of BCR-ABL in this cell line, the dose-dependent inhibition curve of Dasatinib:BCR-ABL is transduced through the signaling cascade via MAPK and AKT/mTOR pathway. The schematic is based on kegg pathway hsa05220.*

b) *DecryptM data (dose-response phosphorylation data) of replicates of Dasatinib-treated K562 cells was processed by CurveCurator, and significant phosphorylation sites were identified by different strategies (left: Relevance Score; right: multiple testing corrected p-values using the Benjamini-Hochberg procedure). Each dot is a modified peptide, and the color indicates if a peptide falls into one of the categories (orange: known ABL substrate based on PSP, red: sites with an ABL kinase motif but not established relationship, blue: MAPK-pathway sites based on PSP or KinaseLibrary, yellow: AKT/mTOR-pathway sites based on PSP or KinaseLibrary). Only sites that were deemed significant by either procedure are colored. Thus, gray dots indicate either not-regulated or unknown-regulated.*

c) *A Venn diagram comparing the significant outcome of the relevance score procedure and the Benjamini-Hochberg procedure. Each set was cut with an alpha value of 5%. The fold change asymptote of the relevance score removed 110 significant Benjamini-Hochberg curves because of too low fold change. Conversely, the relevance score could retain 159 curves that possess a big effect size but got lost because of the multiple testing procedure.*

d) *A bar plot displaying the category distributions (red, orange, blue, yellow, and gray) of panel b in the Venn diagram of panel c (unique for the relevance score, intersection, and unique for the Benjamini-Hochberg procedure). The color coding is the same as in panel b. The explained ratio measures the proportion of annotated sites for each subset.*

Source data for panels b-d are provided as a Source Data file.

Supplementary Notes:

In this section, we describe the general usage of CurveCurator from the user's perspective based on CurveCurator version 0.2.1. Detailed explanations for the latest version, as well as example parameter files, are available on GitHub. Additional example files, e.g., source data from this manuscript, are available on zenodo. CurveCurator is a general-purpose tool for dose-resolved data analysis. Thus, many options only make sense for some assay types but not for others. Therefore, it is important to understand which options matter for each experimental setup, what the consequences are of some analysis steps, and how results should be interpreted. In the following, we inform users about implementation details and give general recommendations for when to use certain options.

1. Requirements, installation, and execution (page 14)
2. Input and Output (page 15)
3. Run times (page 15)
4. Data preprocessing steps (page 16)
5. 4-parameter log-logistic model estimation (page 18)
6. Evaluating the significance of a dose-response curve (page 19)
7. Thresholding dose-response curves by relevance (page 21)
8. False discovery rate estimation using decoy curves (page 22)
9. MAD-Analysis (page 23)
10. Dashboard (page 23)

1. Requirements, installation, and execution:

CurveCurator is an executable, open-source Python module that can be downloaded from GitHub and PyPI. It can run on any operating system with an installed Python interpreter and a few common open-source libraries (numpy, pandas, scipy, statsmodels, bokeh, toml, tqdm). Installation finishes within minutes. A detailed installation guide is on GitHub.

Standard user: CurveCurator is operated from the shell only. The easiest way to set up the correct environment containing all relevant dependencies is to use the package management and environment management system conda. We provide a step-by-step installation guide on GitHub. The package and its dependencies can be installed with pip: (*pip install curve-curator*). After the installation, CurveCurator can be started from the (anaconda) terminal in three simple steps. First, move into the directory of the data to execute. Then, activate the installed environment called "CurveCuratorEnv" if you follow the GitHub installation guide. Finally, start the pipeline by providing the path to the TOML file of the data and add optional command line arguments, e.g., --fdr or --mad. CurveCurator also provides example datasets to test and explore the pipeline: e.g., decryptM dataset containing ~12k PTM curves based on MaxQuant evidence.txt file. More example files can be found on zenodo.

```
Anaconda Prompt

(base) C:\Users\bflorian>cd ./example_datasets

(base) C:\Users\bflorian\example_datasets>conda activate CurveCuratorEnv

(CurveCuratorEnv) C:\Users\bflorian\example_datasets>CurveCurator .\decryptM_Dasatinib\parameters.toml --fdr --mad
```

Advanced user: CurveCurator can be used as a Python library and thus be integrated into various existing analysis pipelines. To add CurveCurator to your Python standard library and make it importable for any Python script that is in the same environment, simply use pip: (*pip install curve-curator*). Make sure that curve-curator is compatible with the existing environment of your project. After the pip installation, you can make imports and use all functionalities of CurveCurator with the LogisticModel object at its core.

```
In [1]:
import numpy as np
import curve_curator as cc
from curve_curator.models import LogisticModel
```

```
In [2]:
# Create a log-logistic model
lm = LogisticModel(pec50=7.0, slope=1.0, front=1.0, back=0.0)
print(lm)

# Calculate expected responses for a range of doses
log_doses = np.log10([1e-9, 1e-8, 1e-7, 1e-6, 1e-5]) # Molar
y = lm(log_doses)
y

LogisticModel({'pec50': 7.0, 'slope': 1.0, 'front': 1.0, 'back': 0.0})

Out[2]: array([0.99009901, 0.90909091, 0.5       , 0.09090909, 0.00990099])
```

The entire package is fully documented, and most of the source code is unit- and integration-tested to ensure stability and increase robustness for future updates and community collaborations. While the code will be maintained by the authors continuously, we encourage other researchers to contribute to CurveCurator for further standardizing dose-response analyses.

2. Input and Output:

To execute CurveCurator, the user only needs to provide the data file with dose responses and a simple parameter file in TOML notation. In the most general case, any data can be provided as a simple tab-separated file containing the response observations for all doses and a sample identifier column named "Name". Additionally, CurveCurator can directly parse proteomic data on protein- or peptide-level coming from various search engine outputs, including MaxQuant, ProteomeDiscoverer, and DIA-NN. The TOML parameter file contains all relevant information to interpret the input file and run the CurveCurator pipeline. A minimal TOML example file is available, which contains the required options. The user can add optional parameters to the TOML file to adapt the pipeline to the requirements of a specific experiment and data type. Some optional parameters are filled with default values, and other parameters are not used if not provided. Detailed explanations about each TOML parameter and its usage are available on GitHub. We recommend storing each TOML parameter file next to the input and output files to increase the reproducibility and transparency of the analysis.

All output files of CurveCurator will be stored relative to the TOML file by default, but other (absolute) paths can be specified in the TOML file, too. A log file reports each step, pipeline values, total processing times, and potential error messages. The CurveCurator pipeline returns multiple files. The *curves.txt* file contains relevant columns from the input data together with fitted curve parameters, statistics, and classification columns. The *dashboard.html* is an HTML dashboard generated by bokeh that enables interactive exploration of the curve data in the browser, e.g. Firefox, on any computer. For more information regarding browser compatibility, see the bokeh documentation. Selected curves can be exported as figures or data tables (see section "10. Dashboard"). If the user has normalization and/or filters activated, the applied normalization factors are reported for each dose in a file called *normalization_factors.txt* (see section "4. Data preprocessing steps"). If the user has MAD analysis active to detect systematic biases using the <--mad> command line option, the MAD values for each dose are reported in a file called *mad.txt* (see section "9. MAD analysis"). If the user has FDR estimation active using the <--fdr> command line option, a *fdr.txt* file is saved that reports the FDR for the applied decision boundary. Furthermore, all generated decoys are reported in a called *decoys.txt*, which has a similar file format as the *curves.txt* (see section "8. False discovery rate estimation using decoy curves").

3. Run times:

The run time of CurveCurator depends on the size of the data set, fitting mode, fitting speed, size of the decoy curves, and number of cores for parallelization. The following run times were obtained for the Dasatinib example data set (~12,000 curves) on a standard desktop computer (total ~2.5 min) using the standard settings and 5 cores for parallelization:

- *Loading & preprocessing data: 3 s*
- *Fitting curves: ~ 60 s (in parallel)*
- *Simulating decoys: 1 s*
- *Fitting decoys: ~ 60 s (in parallel)*
- *MAD-analysis: 3 s (in parallel)*
- *Thresholding & FDR estimation: 1 s*
- *Rendering Dashboard: 3 s*
- *Saving output: 3 s*

4. Data preprocessing steps:

For raw input types, unique keys need to be provided in the "Name" column. However, for supported omics data types, e.g. proteomics, CurveCurator aggregates the rows by unique genes, proteins, or modified peptides depending on the data type specified in the TOML file. The "N duplicates" column in the curves.txt file reports the number of input rows that were aggregated. Raw values or intensities are aggregated by simply summing the values of the duplicates.

CurveCurator is able to handle missing values (NaNs) in the fitting procedures as well as the statistical evaluation in principle. NaNs do not count as an observed data point because they do not contribute to the variance estimation and the recalibrated F-statistic. The minimum number of valid data points (including the control) is *n=5*. Curves with *n=4* data points will be fitted but not statistically evaluated. Alternatively, curves with too many NaNs could be deemed unreliable and thus be filtered out by setting the NaN-filter in the TOML-file. However, depending on the particular assay type, NaNs could be the consequence of desired depletion, e.g. in affinity competition assays, and thus constitute your most important curves in the data set. In these situations, blindly filtering NaN is not an option. For these cases, CurveCurator supports NaN-imputation if specified by the user in the TOML file. We do not recommend imputing NaNs when these values are missing at random, e.g. in viability data sets, and are difficult to estimate. For omics data, NaNs are typically not missing at random but are the consequence of low-intensity sampling bias and thus can often be imputed with a low-intensity value. The imputed value is drawn from the overall intensity distribution of the data. The user can specify the desired quantile. By default, the 0.5% quantile is used. If users want to apply a more complicated imputation technique, they can do this before the CurveCurator pipeline and disable imputation by CurveCurator. As with any imputation, one should understand the consequences to the dose-response analysis. A static imputation value can artificially lead to lower p-values and alter the curve fold change if many data points are missing for one curve. For each curve, CurveCurator reports how many and which data points were imputed so that users can interpret those curves with caution. Below, we show three curves from the Kinobeads data set (*Kleager et al.*) where imputation was successfully applied to "rescue" the designated targets EGFR for Afatinib and MET for Capmatinib, as well as the off-target EPHA2 for Dasatinib.

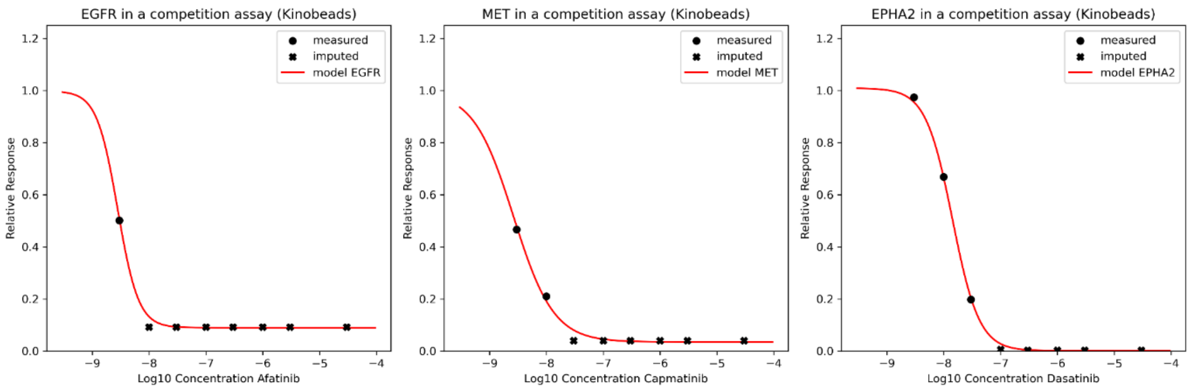

Next, raw intensity values can be globally normalized. This is again an option mostly relevant for omics data sets, where typically the DNA, RNA, or protein distributions stay constant over the course of the treatment, up- and down-regulation are roughly balanced, or regulations do not make a significant proportion (<< 50%) in the data set. Normalization removes channel bias and thus can improve p-values for all truly regulated curves in a data set. However, if one dose is affecting almost all curves in one direction, e.g. toxic concentration in viability assays, normalization can be detrimental to the

analysis because it would remove all biological effects. If the user wants to apply more advanced normalization procedures, it is always possible to do this prior to the CurveCurator pipeline. In the example below (decryptM profiles of Dasatinib in K562 R1), raw intensities were not normalized (left column) or median normalized by CurveCurator (right column) followed by the standard analysis pipeline. In the non-normalized decryptM data, each peptide curve followed the same global trend that was caused by imperfect sample preparation. Removing the systematic variance improved the significance for the example curve ANXA2_pY24 by ~500x times.

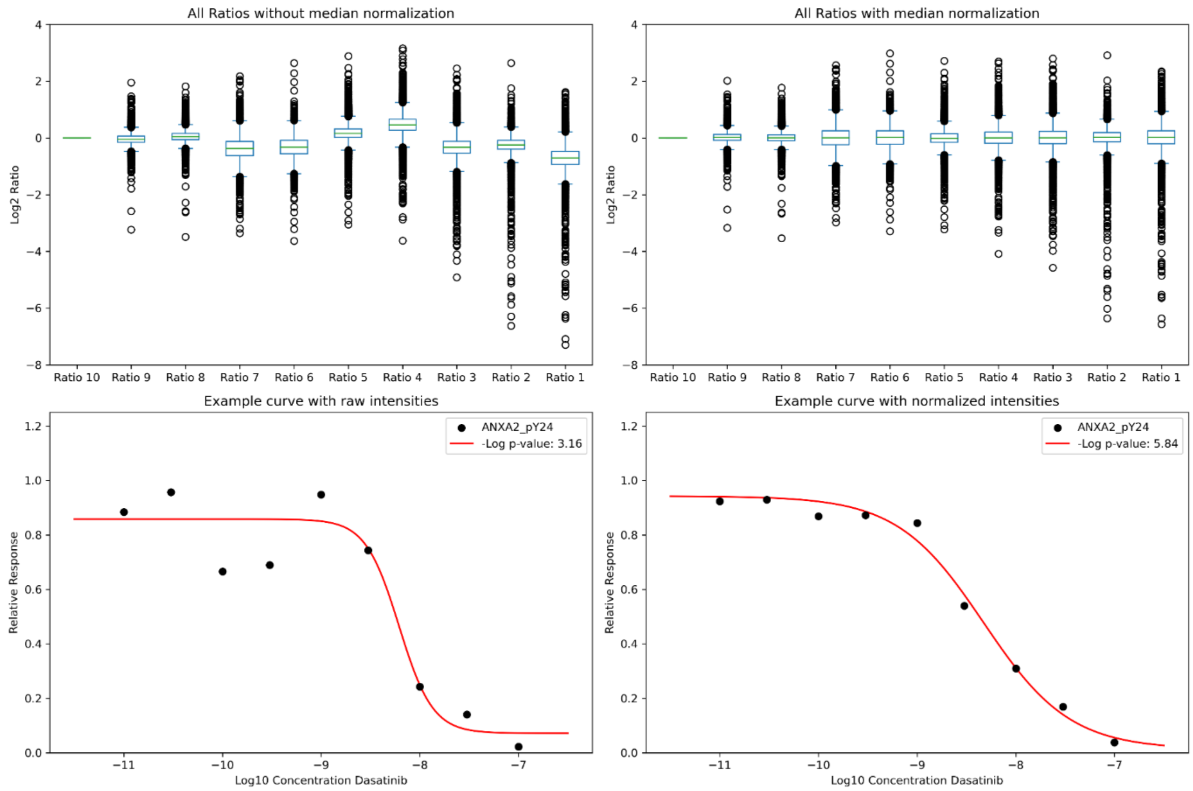

Finally, the processed data is transformed into ratios relative to the control sample. This has the benefit that front and back plateaus are confined to a similar range, e.g. all curves should ideally start at y=1.0, which enables better visualization, fitting, and interpretation of curves. This is especially the case when the absolute value possesses little direct information, e.g. in proteomics, where intensity is proportional to the ions at the detector and not necessarily the absolute moles of a protein / peptide, or in viability assays, where intensity is proportional to some dye concentration in some assay volume as a function of time. It is possible to provide multiple controls to CurveCurator. In this case, the ratios are calculated relative to the mean of the controls. This can improve the accuracy of the front plateau estimate and make the experiment more robust, e.g., in the case that one of the controls has an experimental issue. However, having multiple controls has only little influence on the p-value and the relevance score because the –inf data points do not contain any information about the dose-response relationship.

In the future, we aim to support more software input formats for easier and broader usage of CurveCurator, e.g. dose-resolved transcriptomics. Also, we envision supporting more imputation and normalization strategies to make the tool more versatile.

5. 4-parameter log-logistic model estimation:

CurveCurator uses two competing models, which are evaluated based on the observed responses (normalized to the control sample; see above). To obtain model parameters, CurveCurator currently supports ordinary least squares (OLS) regression as well as maximum likelihood estimation (MLE).

CurveCurator provides different optimization strategies to overcome local minima (Fig. S1), including different initial curve parameter value guesses and multiple minimization rounds. A simple procedure generates alternative initial guesses: i) sort response ratios and doses from lowest to highest dose; ii) set a boundary between each pair of consecutive doses and split the arrays in front (smaller index than the boundary) and back (bigger index than the boundary) points; iii) use a generic log-logistic model for each boundary split by calculating the mean responses for front and back, and using the pEC_{50} value as the middle concentration at the boundary iv) the slope is undefined but can get one or multiple values depending on the fitting mode (see minimization strategies below). In the following figure, different initial guesses were generated along an array of dose-responses. The best starting point is guess #7 because it has the lowest summed squared error.

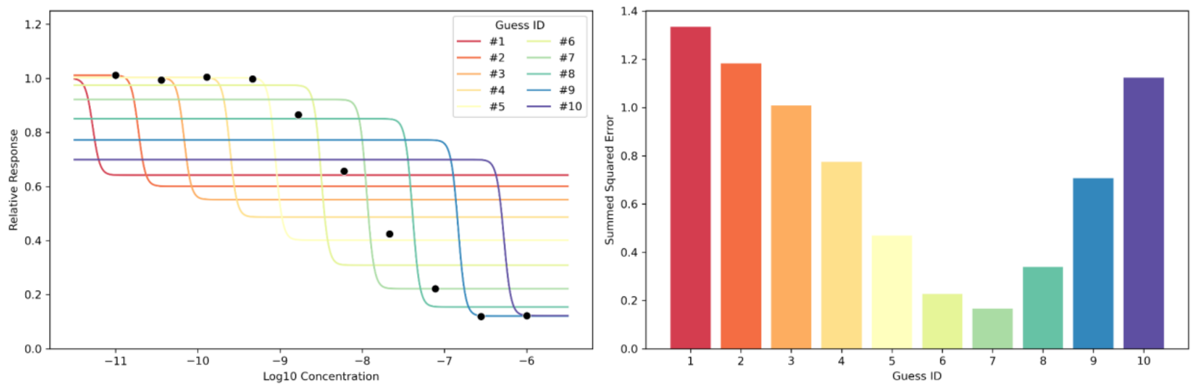

The user can choose from the following four minimization strategies using the *"speed"* parameter in the TOML file (Fig. S2): in "fast" mode, alternative initial guesses are evaluated, and only the best one is used as the initial guess for the minimization; in the "standard" mode, the same best initial guess is evaluated, and then multiple minimizations are performed using different slope parameters together with the best initial parameters; in the "extensive" mode, the entire series of alternative initial guesses is directly minimized; and in the "basin-hopping" mode, the global minimization algorithm "Basin-Hopping" is applied to overcome local minima. If multiple optimization rounds are performed, the fit with the lowest summed squared error after optimization is used.

CurveCurator has many more customizable parameters for the fitting procedure. By default, the following options are not used but can be activated by the user. CurveCurator supports weights in the OLS-fitting approach that can be specified for each data point, which can make curve estimation in some scenarios more robust, e.g, by weighting higher doses more where a stronger and more stable drug effect is expected (for some assays). A subset of the model parameters can also be fixed to a predefined value if the user's experimental setup allows for this assumption, e.g. the back plateau is expected to go to 0.0 for all curves eventually, or the slope is expected to be 1.0 for binary binding events. The optional interpolation method can create more robust fits by linearly interpolating helper points between data points during the fitting procedure, especially when the curve slope is undetermined. These helper points are only used for model estimation. Statistical evaluation is performed solely on real observations. This leads to a slight decrease in explained variance and, thereby, slightly increased p-values.

In the *curves.txt* output file, all parameters related to the dose-response curve (M_1) start with the word "Curve". All parameters related to the Null model (M_0), against which the M_1 is compared, start with the word "Null". Besides the model parameter estimates, CurveCurator provides error intervals, which are based on the scipy implementation, and values derived from the model parameters, for example, area under the curve (AUC), root-mean-squared error (RMSE), or R2.

6. Evaluating the significance of a dose-response curve:

The basic idea behind CurveCurator's recalibrated F-statistics is to evaluate how much better the sigmoidal curve can describe the measured dose responses, and this value needs to be adjusted for the number of data points and number of free parameters. CurveCurator can then translate the F-values to p-values, using a standard F-distribution with optimized parameters to approximate the original simulated distribution (see figure below). A p-value for a curve describes how likely this curve fit resulted in a particular F-value just by random chance. More specifically, a p-value of 0.01 means that 1% of random curves (no response with random errors on the data points) achieved a similar or better F-value than this curve.

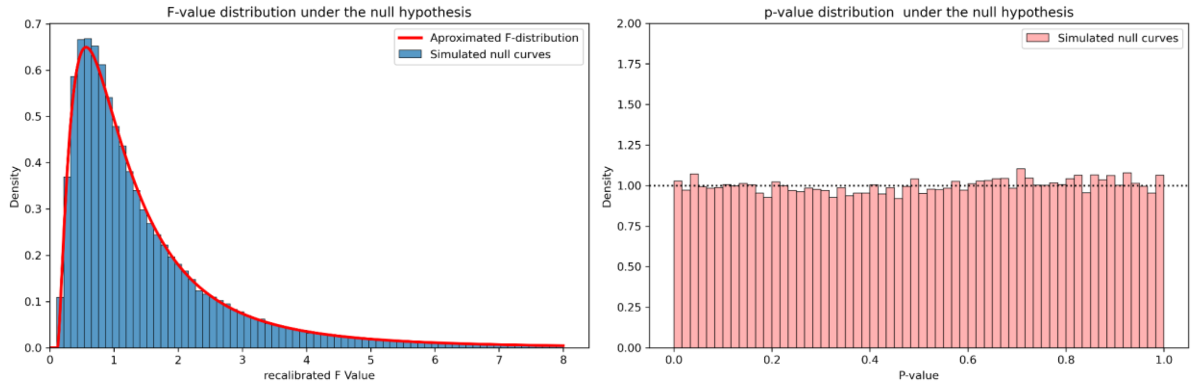

It's important to note that p-values cannot measure if an effect is real or not. It only provides a value reflecting the relationship of variance relative to the observed effect. A high-variance, big-effect curve can have a bigger p-value (=less significant) than a low-variance, small-effect curve just because the model can describe the data points very well. Yet, a low-variance, big-effect curve will produce the smallest p-values (=most significant). Besides variance and effect size, the symmetry of the curve is also relevant to the p-value. It is beneficial to have symmetrical sampling around the expected pEC_{50}, meaning that the front and back plateaus have equally many data points. This is simply rooted in the fact that it is more likely that a single data point is an outlier than many points aligned at the same response height by random chance. In the example below (n=11 data points), different ground truth curves were simulated with low or high variance (rows), small or big effect size (color), and symmetrical or asymmetrical sampling around the pEC_{50} value (columns) to show the effect of the different factors on the p-value.

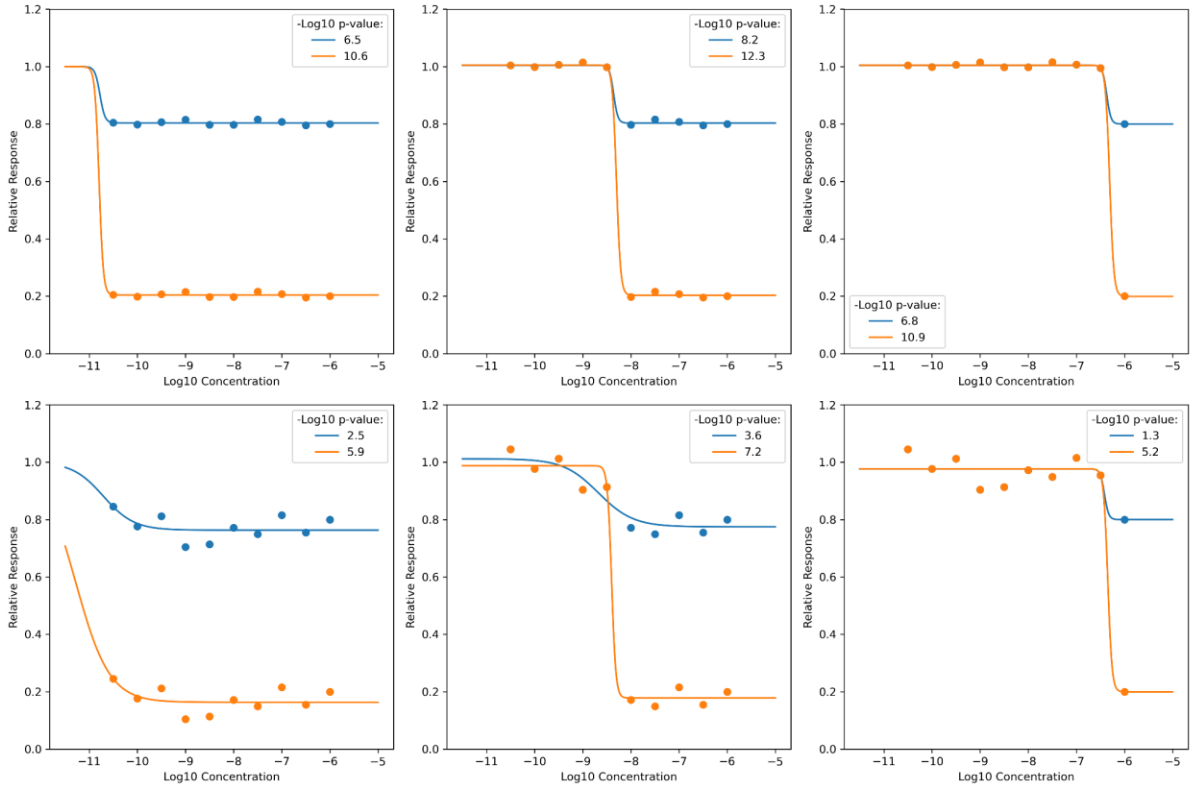

A common mistake in downstream analyses is to treat curve parameter estimates with different confidence as equally reliable. Therefore, we urge users to exclusively interpret the pEC_{50}- values of curves that are deemed significant. Non-significant curves produce random pEC_{50} values along the used dose range. Low potency does **not** indicate curve significance (see figure below for pEC_{50} distribution of random curves from 5 million simulations under the null hypothesis with many high pEC_{50} values). Furthermore, CurveCurator provides standard errors for each curve parameter estimate that can be further used to filter for higher-quality parameter estimates.

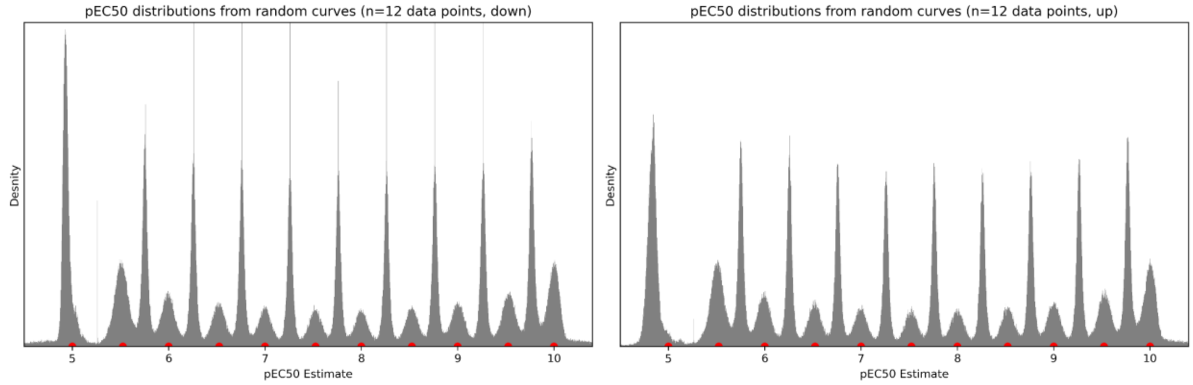

Currently, the calibrated p-values are only supported for the 4-parameter log-logistic model. If some parameters are fixed by the user, e.g. front plateau = 1.0, we cannot guarantee calibrated p-values. However, these p-values are more conservative and would not lead to more false positives. In the future, we aim to provide calibrated p-values for more constrained models as well as more complex models, e.g., the 5-parameter log-logistic model.

7. Thresholding dose-response curves by relevance:

CurveCurator supports users in finding relevant curves. A relevant curve has two properties: i) it is more significant than some alpha asymptote, which controls for random errors, and ii) it has a bigger effect size than some fold change asymptote. CurveCurator transforms this into a continuous function (hyperbolic decision boundary), and users must always provide these two asymptotes for each analysis in the TOML-file. In other words, the alpha-asymptote defines the maximal p-value a curve can have, no matter how big the fold change is, and the fold change asymptote defines the minimal fold change a curve needs, no matter how strong the significance is. Together, this defines the s_0-value (Eq. 12). As a consequence, the position of the hyperbolic decision boundary depends on the two asymptotes and the number of *n* data points (see below).

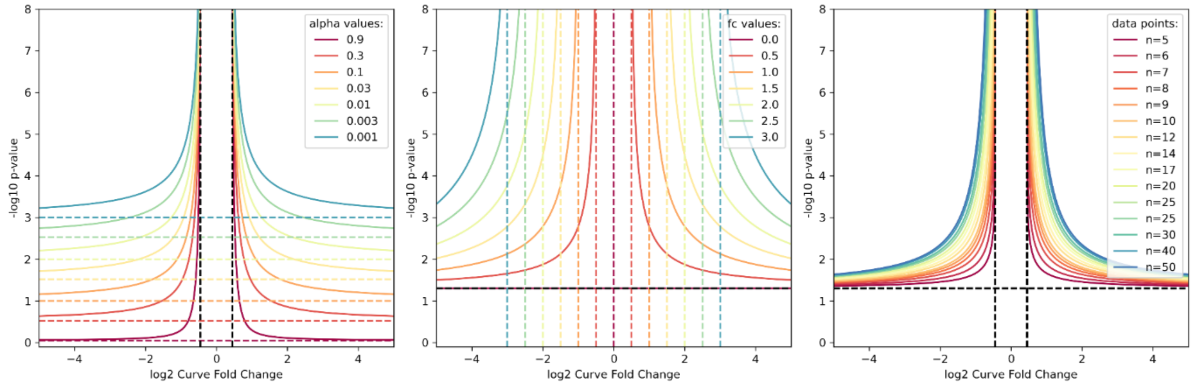

We encourage all users to carefully consider the two asymptote values before the experiment and the latest before the data analysis. The alpha asymptote defines statistical relevance. In practice, the smaller the alpha value, the better the data fits the model and vice versa. Common alpha values are between 0.01 - 0.1, similar to what is often done in differential analysis (e.g., t-tests). We suggest 5% as a good starting point. The fold change asymptote defines biological relevance, which depends on the assay and the biological system in question. As a good starting point, ask yourself: "For this assay, what is the smallest change you would still follow up on?" Since the two asymptotes define a robust decision boundary, there will not be a considerable difference between very close values. This, however, does not mean that the value is not important. A well-chosen fold change asymptote can provide a lot of power to the analysis. A second consideration for the fold change asymptote relates to the overall log_2 ratio variance of the assay. If the assay is generally not very precise, the fold change asymptote should be increased as the random curves (as well as the decoys) can spread over a wider range of fold changes. Typically, this is also indicated by a surprisingly high FDR and becomes more and more problematic when fewer data points exist per curve. The opposite is true for assays with very little variance that can even capture subtle differences reliably. Here, the fold change asymptote could be even loosened if desired.

In the following, we explain the asymptote choices for specific datasets used in the manuscript :

<u>Kinobeads</u>: This is an in-vitro drug-target competition pulldown assay with MS-readout using a complex cellular lysate and ATP-pocket-binding affinity matrices. Ideally, the drug completely blocks true targets (pre-incubated), and thus the protein signal should vanish. Consequently, a relatively strong log2 fold change cutoff of 0.5 was chosen. Since the thresholds define a hyperbolic decision boundary, most curves pass a threshold in the "hinge region" (marked in the left picture below) where the log2 fold change is around ~1.3, corresponding to a curve that goes down by 60%. This strong fold change cutoff entails a big reduction of the FDR, which allowed us to lower the alpha

value to 10%. In the right plot, the manually expert-selected kinase targets were highlighted, indicating that the chosen decision boundary matches the initial manual expert curation well.

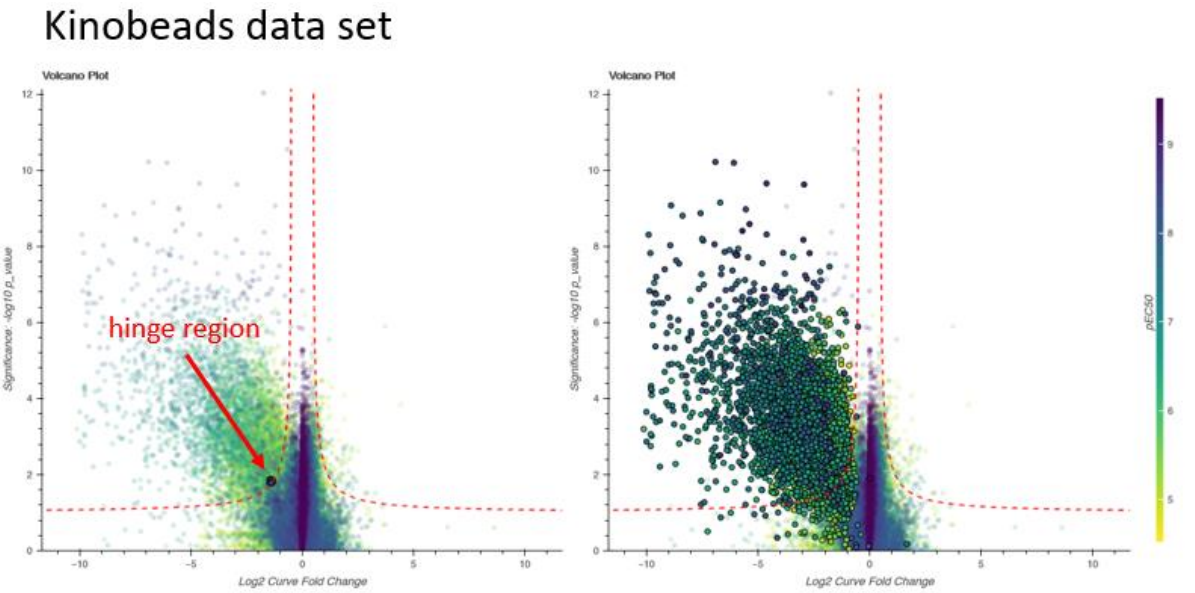

CTRP Viability data: This is a CellTiter-Glo assay measuring ATP levels in cells, capturing both growth inhibition and potential cell toxicity at a population level. The authors were impressed by the high quality of the data set (very low noise, 16 data points), especially when comparing this data against other publicly available viability data sets. We chose the default alpha asymptote of 5%. For the fold change asymptote, we used a value of 0.3, which results in a rather permissive fold change asymptote. The reason for this was to retain curves even when they only slightly changed population growth behavior (e.g., mTOR inhibitor Rapamycin; see figure below). This was only possible because the data quality of this data set was very high. If the goal was to identify drug-killing curves, one would choose a much stronger fold change limit instead. This highlights the importance of understanding the goal of an analysis, and it is up to the user to know what their goal is.

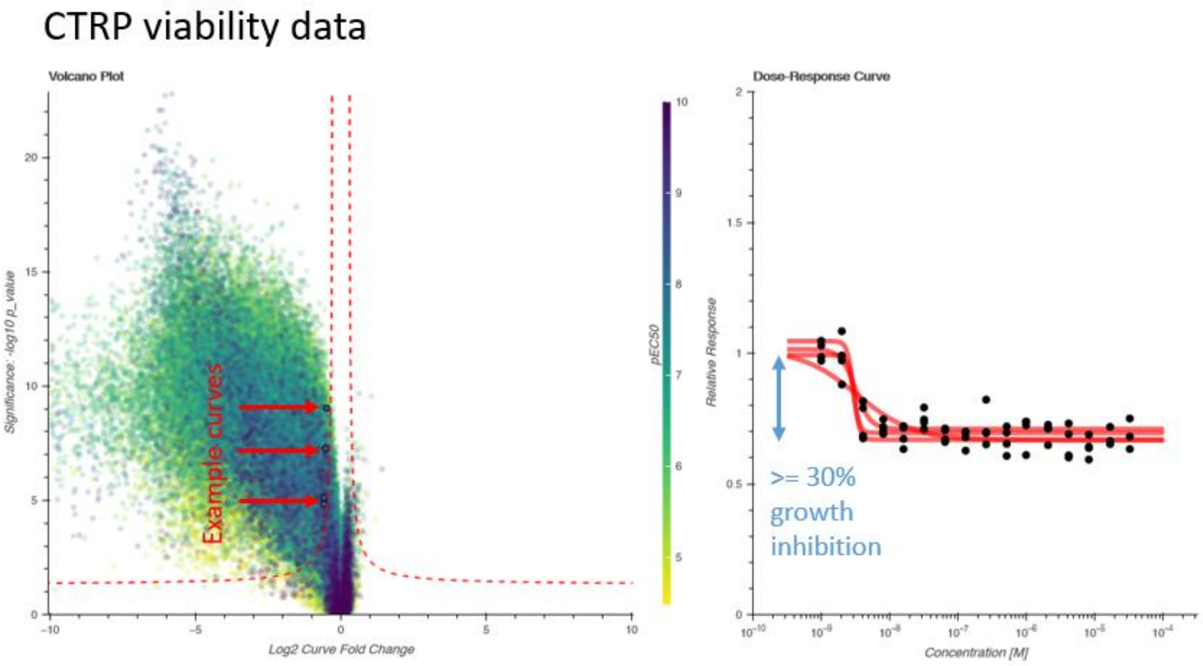

decryptM: This is an in-cellulo drug perturbation assay with a mass-spectrometry-based readout of changes in post-translational modifications (PTMs). Here, even when using TMT-labeling to reduce workflow variance and increase quantitative precision, our experience is that different phosphorylation sites can span a wide range of variances, unlike in a typical cell viability assay where one can assume a constant random assay error. Again, we took the default 5% alpha asymptote. For the log2 fold change asymptote, we chose a relatively conservative value of 0.45, which results in a log2 fold change of ~2 in the hinge region of the decision boundary.

The Relevance Score is an alternative representation of the hyperbolic decision boundary. Instead of drawing the boundary in two dimensions, the s_0-value is used to adjust each curve's F-value by its effect size (Eq. 13). As a consequence, curves with big effect sizes are less affected by the adjustment than curves with small effect sizes. From a graphical representation, it's related to the distance to the fold change asymptote. In the example figure below, the red curves have different fold changes or p-values but have the same biological relevance based on the user's initial definition of relevance. The purple and blue curves have the same significance (p-value) but have different relevance because of their different fold changes. The asymptotes are represented as dotted lines, and the hyperbolic decision boundary is an unbroken line (before and after the s_0-transformation).

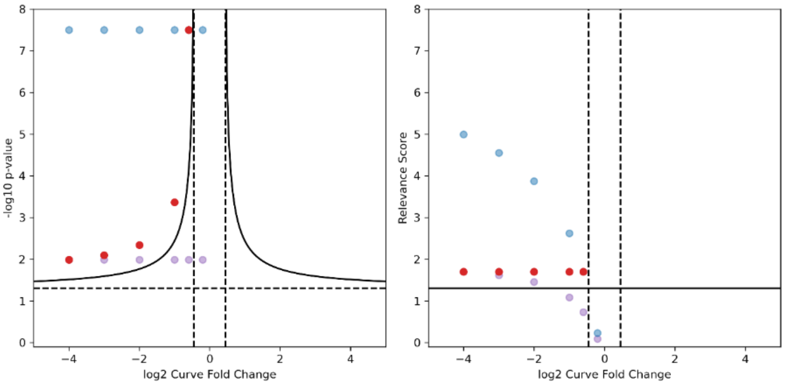

Users can opt not to consider fold changes by simply setting fc_{lim} = 0.0. Consequently, there will only be a horizontal decision boundary, and relevance is purely defined by significance. In these cases, p-values need to be corrected for multiple testing. This can be done by choosing a desired technique (mtc_method in TOML file), e.g., the Benjamini-Hochberg procedure. Various procedures are based on available methods from statsmodels. It is also possible to combine multiple testing corrections with an additional fold change limit, which will result in the poorest possible combination regarding performance. Here, p-values are first multiple testing corrected and then additionally cut by a fold change threshold. We recommend the default s_0-based approach, which shows the best performance.

8. False discovery rate estimation using decoy curves:

Because CurveCurator identifies relevant curves using the relevance score, which is, although related, not a p-value, so a different method is necessary to estimate the number of false positives for a given data set and a given decision boundary. CurveCurator uses a target-decoy approach to do this. All experimentally measured dose-response curves are considered target curves, no matter if they are significant or not. Based on the error around the target curves, CurveCurator can estimate the underlying variance distribution of a data set (Eq. 15). Please note that each data set has a different variance distribution, which affects how high of an effect size a random curve can obtain. Using the

estimated empirical variance distribution, decoys are generated. A decoy curve models no response (=response is independent of the dose) around the relative ratio 1.0. All deviations from 1.0 are from random errors based on a draw from the empirical variance distribution of the targets (Eq. 1). All decoys are then fitted using the same conditions as for the targets. This obtains an adjusted F-value and relevance score distribution for targets and decoys. CurveCurator can now apply a single relevance score threshold, and the number of significant decoys reflects the number of expected false positives in the data set from which the false discovery rate is calculated. Furthermore, a q-value for each target curve is calculated during the target-decoy competition.

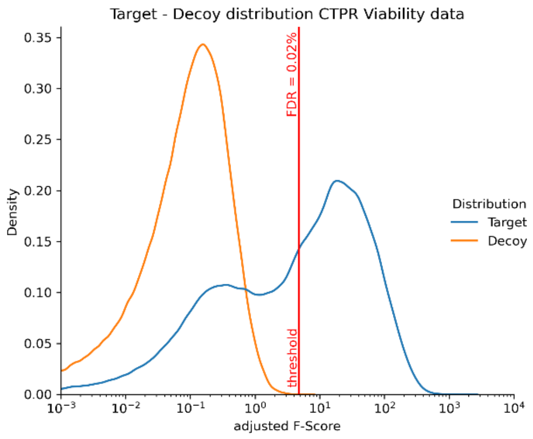

With the decoy ratio parameter, users can set how many decoys should be used to estimate the FDR. The more decoys are simulated, the more accurate the FDR estimation will be. However, the overall run time increases the more decoys are fitted. The FDR is also more accurately estimated when more data points per curve exist because this allows a more accurate estimation of the true underlying variance distribution.

CurveCurator is deliberately not supporting cutting curves by FDR. The reason for this is that i) the fold change asymptote alone is often a very strong threshold, and ii) the previous user-defined asymptotes will change (at constant s_0) when cutting by an FDR threshold.

9. MAD-Analysis:

CurveCurator is able to detect systematic problems in data sets. This is particularly relevant for proteomics data, where one raw file or TMT channel can exhibit disproportionately high variance and interfere with the statistical evaluation. The median absolute deviation (MAD) analysis is a simple way of detecting aberrant variance by looking at residual distributions over all curves in the dataset. A MAD-value of, e.g., 0.1 means that 50% of all curves are further away from the model estimate than 0.1 ratio units. For proteomics data, we consider high-quality data to be <0.1 and exclude data points if 0.15 is exceeded. As most measurements are close to 1, these values can be interpreted as 10% and 15% variance thresholds. Users can activate the MAD analysis via the <--mad> command line option.

10. Dashboard:

CurveCurator outputs an interactive dashboard that allows for fast and interactive data exploration. All data is stored inside the HTML, which can be opened by a variety of browsers. This makes data

visualization, data sharing, and collaboration easy as files can be shared by email and does not require any server system.

Depending on the assay type, different options are available. All dashboards have a global overview plot on the left side, which can switch between volcano and potency representation. Each dot is one dose-response curve, and the color indicates its potency, which can be decoded with the color bar next to the global plot. The volcano plot view relates the curve fold change to the curve significance (p-value) or relevance score. The red dashed line indicates the decision boundary line that was set in the TOML file via the fold-change and alpha asymptotes. The potency plot view relates the curve fold change to the curve potency measured by pEC_{50}.

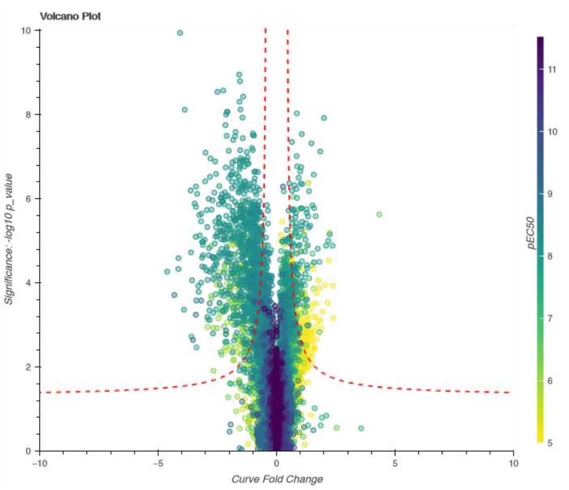

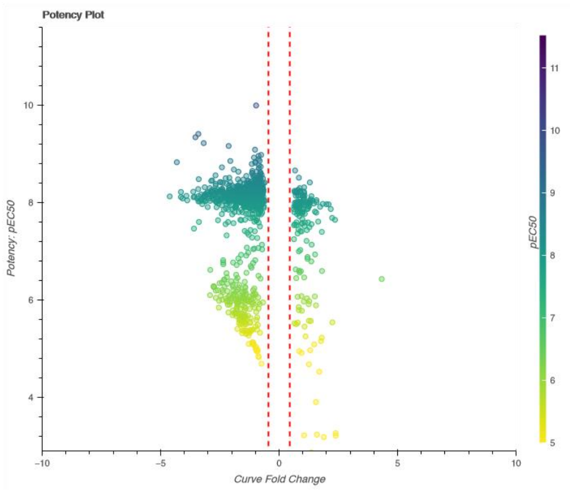

The global overview plot can be controlled by the top menu bar:

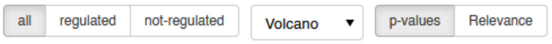

The default setting, when loading or refreshing the page, shows all curves in a volcano plot with p-values as y-axis (figure above). The button options "all", "regulated", and "not-regulated" will hide/show curves based on CurveCurator's curve classification. The different data selections can be activated by clicking on the button. The next selection can switch between the volcano or potency view on the data. Only in the volcano plot view, different y-axis options are available (p-values, relevance score, corrected p-values). Furthermore, the dataset can be subselected by applying range filters and only curves in between the sliders are visualized:

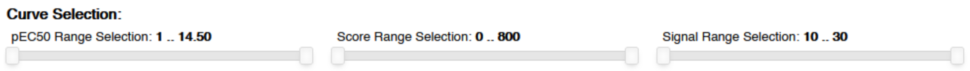

The global plots are interactive and can be controlled with the mouse. The dashboard offers different options to interact with the plot, which can be selected on the top right corner of the global plot:

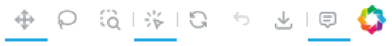

By clicking on the tools, one can activate or deactivate the tool. Activity is indicated by the blue line under the icon, which modifies the behavior of the mouse. With "Pan", one can move the plot around. With "Lasso" and "Tap", one can select multiple curves. With "Box Zoom", one can zoom in on a certain area of interest. With "Reset" and "Undo", one can revert changes made to the plot without refreshing

the page. The "Save" button will save the current plot as a PNG or SVG file, depending on the backend mode. The "Hover" option will show a small information box next to the curve for each curve where the mouse is currently located. Furthermore, bokeh offers a variety of keyboard shortcuts to interact with the plots more efficiently. For more information, see the bokeh documentation.

By selecting one or multiple dots (=curves), the dose-response area visualizes the selected curves and yields a quick overview of the data points and the fitted curve(s). More information about selected curves can be found at the bottom table. By clicking on the headers of the table, rows can be sorted alphabetically or numerically. Table elements can be marked and copy-pasted to other programs.

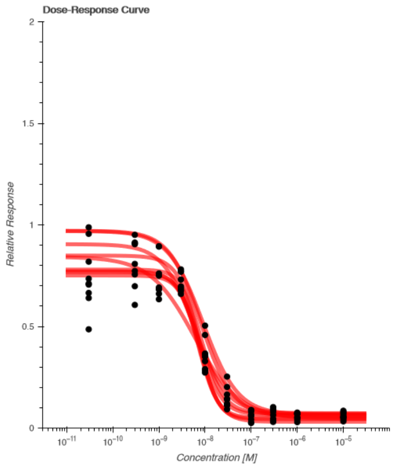

#	Name ▲	Modified Sequence	Gene(s)	Uniprot ID(s)	Score	Curve pEC50 [M]	Slope	Fold change	AUC	R2	Relevance Score	Signal Quality
11634	ARHGEF1	EPLLSS(ph)SENGNGGR	ARHGEF1	M0QZR4;Q92888-2;Q92888;Q92888-3;	252.68	8.114	2.254	-4.144	0.365	0.803	3.466	16.932
11703	DDI2	LFS(ph)ADPFDLEAQAK	DDI2	Q5TDH0-2;Q5TDH0;Q5TDH0-3;H0YII4	367.05	8.126	1.96	-4.617	0.35	0.792	3.497	20.737
11753	DDRGK1	RLES(ph)QR	DDRGK1	A0A0A0MRX2;Q96HY6-2;Q96HY6	110.87	8.139	2.121	-3.912	0.353	0.634	2.722	16.313
11622	FAM83H	LSS(ph)ATANALYSSNLR	FAM83H	Q6ZRV2;A0A494C1T9;J3KPS2	465.65	8.111	1.722	-3.61	0.41	0.858	3.626	21.287
11212	MAPK1	VADPDHDHTGFLT(ph)EY(ph)VATR	MAPK1	P28482-2;P28482	327.81	8.045	1.15	-3.877	0.471	0.986	5.53	17.953
11487	MAPK3	IADPEHDHTGFLT(ph)EY(ph)VATR	MAPK3	B3KR49;E9PJF0;E9PQW4;P27361-2;P	191.5	8.086	1.259	-4.076	0.461	0.996	6.249	17.35
11647	MISP	HLS(ph)ESSGKPLSTK	MISP	Q8IVT2	141.99	8.117	1.834	-3.57	0.373	0.792	3.169	22.038
121...	SLC9A7	TKS(ph)SSEEVLER	SLC9A7	A0A087WXD1;Q96T83	211.57	8.223	1.254	-3.623	0.42	0.948	4.516	19.034
121...	TPR;SACS	LQT(ph)TLR	TPR;SACS	P12270;Q9NZJ4-2;Q9NZJ4	95.815	8.218	0.898	-4.142	0.379	0.86	3.648	15.839

The search boxes enable complex data queries and are fully regex compatible. For example, one can look for one drug and highlight all sensitive cell lines in a viability data set or even a generalized kinase motif in phospho data (see figures below).

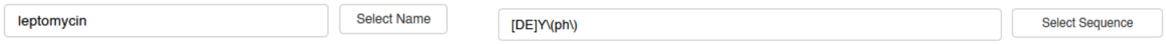

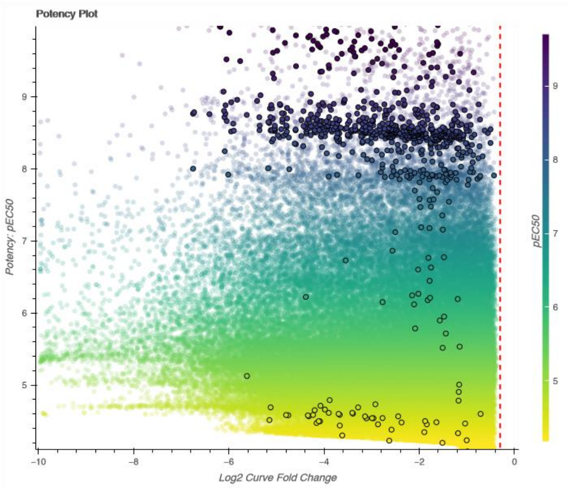

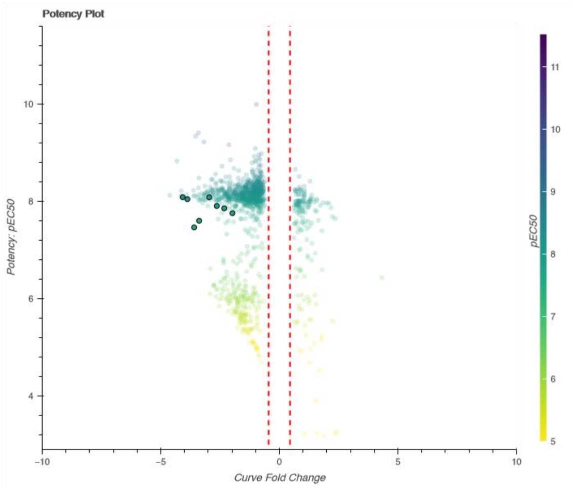

Below are two screen shots of the tool in action:

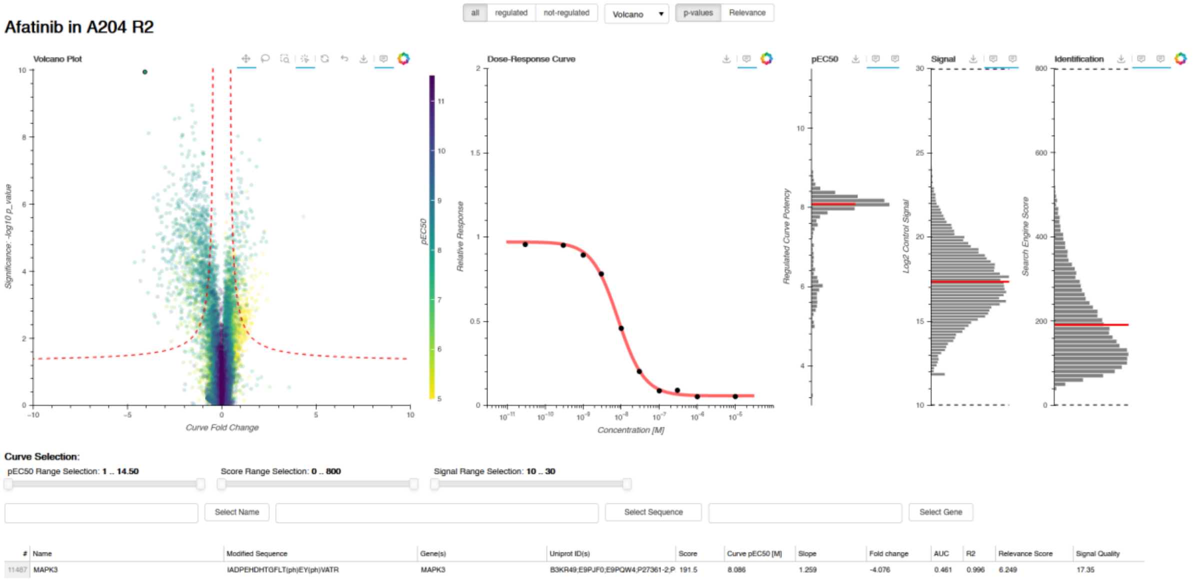

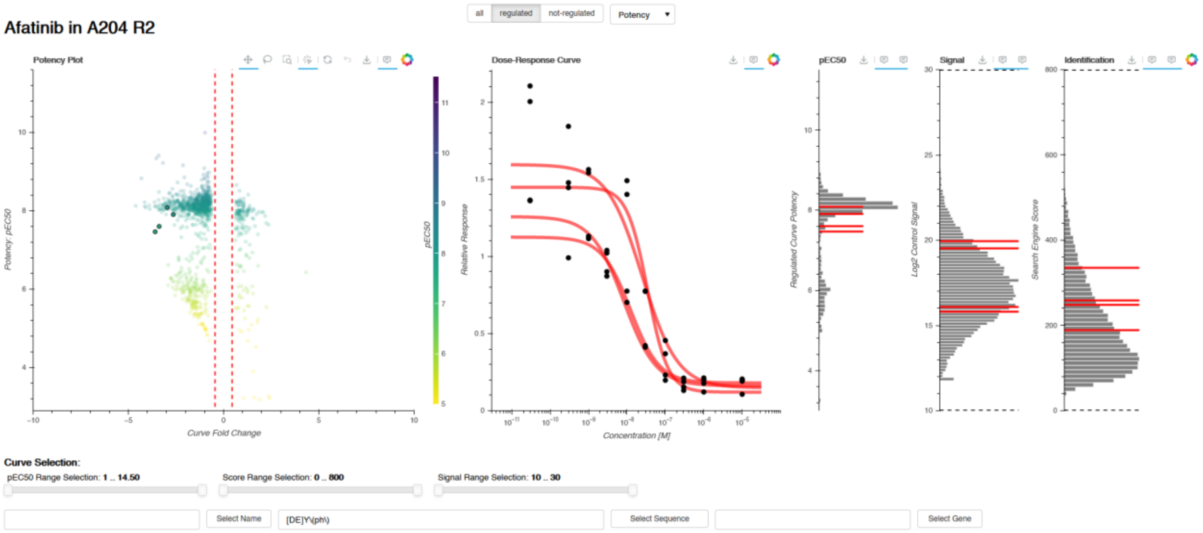

#	Name	Modified Sequence	Gene(s)	Uniprot ID(s)	Score	Curve pEC50 [M]	Slope	Fold change	AUC	R2	Relevance Score	Signal Quality
8108	EGFR	GSHQISLDNPDY(ph)QQDFFPK	EGFR	E9PFD7;P00533	335.29	7.465	1.629	-3.592	0.856	0.198	2.192	19.521
8662	EGFR	GSTAENAEY(ph)LR	EGFR	E9PFD7;P00533	248.15	7.602	0.948	-3.383	0.913	0.076	1.968	19.939
100...	GAB1	QVEY(ph)LDLDLDSGK	GAB1	Q13480-2;B7Z3B9;Q13480	258.65	7.905	1.157	-2.64	0.628	0.846	3.13	16.085
11481	GAB1	VDY(ph)VVVDQQK	GAB1	Q13480-2;B7Z3B9;Q13480	188.87	8.084	1.035	-2.959	0.645	0.792	3.171	15.803
