# Supplementary material for: CurveCurator: a recalibrated F-statistic to assess, classify, and explore significance of dose–response curves
Source: Nat Commun. 2023 Nov 30;14:7902. doi: 10.1038/s41467-023-43696-z (PMC10689459; doi:10.1038/s41467-023-43696-z)
Supplement: Supplementary file 3 — Description of Additional Supplementary Files [file 41467_2023_43696_MOESM3_ESM.pdf]

Description of Additional Supplementary Files:

Supplementary Dataset 1: Comparison of the fitted curves by CurveCurator and the original manual classification (Klaeger et al. 2017) for the Kinobeads dataset.
